# Incidence and factors associated with prolonged use of mechanical ventilation in pediatric intensive care unit in a single tertiary care hospital

**DOI:** 10.1371/journal.pone.0311275

**Published:** 2024-11-11

**Authors:** Varisa Pisitcholakarn, Kanokkarn Sunkonkit, Sanit Reungrongrat

**Affiliations:** 1 Department of Pediatrics, Faculty of Medicine, Chiang Mai University, Chiang Mai, Thailand; 2 Division of Pulmonology and Sleep Medicine, Department of Pediatrics, Faculty of Medicine, Chiang Mai University, Chiang Mai, Thailand; University of Cape Town, SOUTH AFRICA

## Abstract

**Introduction:**

Invasive mechanical ventilation (IMV) is frequently used as a life-supporting device in Pediatric Intensive Care Units (PICU). To date, there are few studies evaluating the impact of prolonged mechanical ventilation (PMV) in children which is associated with high morbidity and mortality. We aimed to determine the incidence and factors associated with PMV in PICU at our institution.

**Methods:**

A retrospective review was performed of children aged 1 month to 18 years who were admitted to the PICU at Chiang Mai University Hospital, Thailand between January and December 2020. PMV was defined if the duration of IMV was ≥ 96 hours. Baseline characteristics and factors associated with PMV were analyzed by descriptive statistics, and univariable and multivariable logistic regression analysis, respectively. A *p*-value of < 0.05 was considered significant.

**Results:**

Ninety-two episodes of IMV were performed in 90 children. The median (IQR) age of the children was 22.8 (7.2–111.9) months (male 64.1%). Forty-six of 92 (50%) children received PMV and 32.6% of children with PMV required a tracheostomy. Following multivariable analysis, factors associated with PMV were age <2 years old (OR 2.86, 95% CI 1.04–7.84, *p = 0*.*041*), male gender (OR 3.21, 95% CI 1.15–8.94, *p = 0*.*026*), and multiple antibiotics administration during PICU admission (OR 7.83, 95% CI 1.87–32.78, *p = 0*.*005*), respectively.

**Conclusions:**

Pediatric PMV was notably common, with younger age, male gender, and multiple antibiotic use contributing to higher risk. Developing weaning protocols and strategies to reduce PMV duration is crucial.

## Introduction

Mechanical ventilation (MV), a life-supporting invasive device in the intensive care unit (ICU), helps to improve oxygenation and ventilation as well as decrease the work of breathing. Invasive mechanical ventilation (IMV) is generally required in approximately 35 to 64% of ICU patients [1]. Similarly, several studies demonstrated that children who have been on MV range from 17–64% in developed countries and 30–52% in developing countries, respectively [2, 3]. However, the duration of IMV is varied and dependent on diagnosis, condition, and underlying disease of the children [1–4].

According to the International Classification of Disease (ICD), 9^th^ Revision, Clinical modification of the United States, prolonged mechanical ventilator (PMV) is defined as MV use for at least 96 hours [5]. Some studies found that the increased MV duration is associated with higher morbidity and mortality [6, 7]. In addition, PMV is associated with longer lengths of hospital stay and higher health care costs [6–9]. The incidence of PMV and mortality in adult ICUs ranged from 6 to 9% and 24 to 65%, respectively [10–13]. The risk factors associated with PMV in adult ICUs were old age, the severity of illness, the presence of chronic comorbidities, hypokalemia, hypophosphatemia and hypoalbuminemia [13–19]. However, the studies regarding the incidence and factors associated with PMV in children have been limited, to date [10, 20, 21]. Some factors associated with PMV in children were the use of non-invasive ventilation (NIV) before intubation, continuous intravenous sedation on the first day of ventilation, multiple antibiotics, vasoactive drug support, higher ventilatory parameters and ventilator-associated pneumonia (VAP) which differed from adult studies [10, 16, 22].

The factors linked to PMV in children are crucial for enhancing therapeutic strategies in PICU care. This study aimed to identify the incidence and outcomes of PMV, along with the associated factors, to guide immediate medical decisions and improve PICU management for best practices.

## Materials and methods

### Study population

Electronic Patient Charts from PICU at Chiang Mai University Hospital, Thailand were retrospectively reviewed on children aged 1 month to 18 years who were admitted and received IMV between January and December 2020. Our PICU admits pediatric patients with both medical and surgical conditions. Medical conditions managed include respiratory failure, ARDS, septic shock, renal failure requiring renal replacement therapy, liver failure, neurological and hematologic conditions, as well as transplantation, such as liver and stem cell transplantation. For surgical conditions, the PICU handles non-cardiac postoperative care. Children with congenital heart disease (CHD) and those who have undergone cardiac surgery receive ICU care in a separate unit. This study was approved by the Research Ethics Committee of the institution (IRB No. 125/2564). The data were accessed for research purposes during April, 2021 to January, 2022.

We defined PMV as MV through an endotracheal tube or tracheostomy for at least 96 hours or more. Non-PMV was defined if the duration of MV use was less than 96 hours. The cut point of timing (96 hours) was used based on the ICD, 9^th^ Revision, Clinical modification [5].

The timing of each episode of MV was defined as the time from intubation to extubation. If children were reintubated within 48 hours after extubation, the episode was classified as the same case. In contrast, children who were reintubated after extubation after more than 48 hours were classified as having different MV episodes.

### Inclusion criteria

Children aged 1 month to 18 years who were admitted and received IMV in PICU at Chiang Mai University Hospital, Thailand during the study period were included in the study.

### Exclusion criteria

1. Children diagnosed with CHD and those requiring cardiac surgery were excluded from the study, as these patients were admitted to a separate ICU unit in our hospital.
2. Children who were diagnosed as brain dead before PICU admission and had been prescribed IMV for more than 48 hours prior to PICU admission
3. Children with tracheostomy and home ventilation who had undergone tracheostomy prior to PICU admission

### Data collection

Data were gathered by reviewing the medical record from the Digicard Program of Chiang Mai University Institute, Thailand. The data were collected including demographic data, laboratory investigations, and maximal ventilatory parameters during 24 hours of admission, treatment, and complications during 96 hours of admission, as well as the outcome of treatment.

Demographic data were recorded at PICU admission including age, gender, weight, nationality, Pediatric Index of Mortality 2 (PIM-2) at PICU admission, NIV use before intubation, comorbidities, and admission reasons which were classified into 6 categories; 1) respiratory failure due to severe pneumonia or acute respiratory distress syndrome (ARDS); 2) postoperative care; 3) respiratory failure due to neurological conditions; 4) respiratory failure due to septic shock; 5) respiratory failure due to bronchopulmonary dysplasia (BPD) exacerbation; and 6) respiratory failure due to asthmatic exacerbation.

During 24 hours of admission, laboratory investigations and ventilator parameters were recorded. The laboratory investigations included 1) hypokalemia (potassium less than 3.5 mmol/L); 2) hypophosphatemia (phosphorus less than 3.5 mg/dl); 3) hyponatremia (sodium less than 135 mmol/L); 4) hypoalbuminemia (albumin less than 3 g/dl); and 5) hemoglobin (g/dl). Maximal ventilatory parameters included peak inspiratory pressure (PIP), positive end-expiratory pressure (PEEP), the fraction of inspired oxygen (FiO_2_), and mean airway pressure (MAP).

During 96 hours of invasive ventilation, treatment, and complications were documented. The treatment included 1) red blood cell transfusion ≥ 10 ml/kg; 2) multiple antibiotics (≥ 2 types of antibiotics); 3) corticosteroids use; 4) continuous sedation on the first day of admission; and 5) vasoactive drug use. Moreover, the complications consisted of 1) VAP (defined by the Centers for Disease Control (CDC) and Prevention diagnostic criteria, namely temperature of > 38.5 c or < 36 c, white blood cell count (WBC) of > 12,000 or < 4,000 cells/mm^3^, purulent bronchial secretions and alveolar infiltrates) [23]; 2) urinary tract infection (UTI) (defined by WBC > 5/HPF on urinalysis and positive urine culture: any growth by suprapubic aspiration, >5 × 10^4^ CFU/ml by urethral catheterization, or >10^5^ CFU/ml by midstream clean catch) [24]; 3) central line-associated bloodstream infection (CLABSI) (defined by a laboratory-confirmed bloodstream infection (LCBI) where an eligible bloodstream infection organism is identified, and an eligible central line is present on the LCBI date of the event or the day before) [25]; and 4) multiple organ failure (≥ 2 systems) (each organ dysfunction criteria was based on the International Pediatric Sepsis Consensus Conference Criteria) [26].

The outcome of treatment was collected as the length of stays (LOS) in the hospital, LOS in PICU, and mortality in PICU.

### Statistical analysis

The baseline characteristics were summarized using descriptive statistics. Descriptive statistics included frequencies, percentages, and mean (standard deviation; SD) or median (interquartile range; IQR) as appropriate will be used to summarize the study population. The incidence of PMV was calculated using the number of PMV admissions divided by the number of IMV admissions during the study period. The parameters were compared using the χ2 test for categorical variables and one-way ANOVA for continuous variables. The factors associated with PMV were analyzed by univariable and multivariable logistic regression analysis. A collinearity diagnosis was conducted to assess for multicollinearity between variables. A variance inflation factor (VIF) between 0 and 10 suggested the absence of collinearity. Data were analyzed using SPSS version 26 (IBM Corp, Armonk, NY. Significance was determined as a p-value of *<0*.*05*.

## Results

One hundred and sixty-three children were admitted to PICU. Of these, sixty-eight children were not intubated during PICU admission due to NIV use (n = 60), plasma exchange (n = 4), stem cell transplantation (n = 3), requiring continuous renal replacement therapy (CRRT) (n = 1). Of the 95 children who were prescribed IMV, 5 children were excluded as follows; 4 children required IMV more than 48 hours before PICU admission and the other patient were > 18 years of age. Ninety-two episodes of IMV were performed in 90 children (male 64.1%). Forty-six of 92 (50%) episodes required MV for ≥ 96 hours which were assigned to the PMV group. The median (IQR) length of mechanical ventilation was 95.5 (30.5, 204.0) hours after intubation. The primary indication for IMV upon admission to the PICU was acute respiratory failure resulting from severe pneumonia or ARDS, accounting for 47.8% of cases. Out of the 92 children who were prescribed IMV, 6 (6.5%) were diagnosed with neutropenia at the time of admission. Fifteen of 46 (32.6%) children in the PMV group required IMV underwent tracheotomy due to extubation failure, with neurological conditions accounting for 80% (12/15) and respiratory conditions for 20% (3/15).

A significantly higher rate of multiple antibiotics 93.5% (43/46), continuous sedation on the first day of PICU admission 30.4% (14/46), and vasoactive drug use 41.3% (19/46) in PMV group compared to the non-PMV group were noted. VAP and UTI complications occurred within the first 96 hours of PICU admission and were significantly more frequent in the PMV group compared to the non-PMV group. No cases of CLABSI were observed during the study period. Among the 15 patients who developed VAP, the respiratory pathogens identified were as follows: *Acinetobacter baumannii* in 10 patients (66.7%), *Candida albicans* in 1 patient (10.0%), *Pseudomonas aeruginosa* in 1 patient (10.0%), a mixed infection of *Acinetobacter baumannii* and *Pseudomonas aeruginosa* in 2 patients (20.0%), and a mixed infection of *Acinetobacter baumannii* and *Klebsiella pneumoniae* in 1 patient (10.0%). The mortality rate in our PICU cohort was 14.1%. Table 1 presents the baseline characteristics of the study population.

**Table 1:** Baseline characteristics at PICU admission (n = 92).

Characteristics		Total	PMV	Non-PMV	P-value
		N = 92	N = 46	N = 46	
**Age, months**		22.8 (7.2, 111.9)^#^	17.4 (7.2, 64.2)^#^	29.4 (9.0, 141.6)^#^	*0*.023*
**Male, n (%)**		59 (64.1)	24 (52.2)	35 (76.1)	*0*.029*
**BMI, kg/m** ^**2**^		15.8 (14.1, 17.8)^#^	15.6 (13.7, 17.4)^#^	16.3 (14.4, 18.2)^#^	0.401
**Comorbidity**	**History of prematurity, n (%)**	20 (21.7)	13 (28.3)	7 (15.2)	0.206
	**Respiratory disease, n (%)**	20 (21.7)	14 (30.4)	6 (13.0)	0.075
	**Neurologic disease, n (%)**	21 (22.8)	8 (17.4)	13 (28.3)	0.321
	**Hematologic disease, n (%)**	17 (18.5)	10 (21.7)	7 (15.2)	0.592
	**Cancer, n (%)**	4 (4.4)	0	4 (8.7)	0.117
	**Metabolic/endocrine disease, n (%)**	3 (3.3)	2 (4.4)	1 (2.2)	1.000
	**GI disease, n (%)**	18 (19.6)	12 (26.1)	6 (13.0)	0.188
	**Multiple anomalies, n (%)**	10 (10.9)	7 (15.2)	3 (6.5)	0.315
	**Other comorbidities, n (%)**	9 (9.8)	3 (6.5)	6 (13.0)	0.485
**Admission reason, n (%)**	**Respiratory failure due to severe pneumonia or ARDS, n (%)**	44 (47.8)	28 (60.9)	16 (34.8)	*0*.021*
	**Postoperative care, n (%)**	28 (30.4)	5 (10.9)	23 (50.0)	*<0*.001*
	**Respiratory failure due to neurological conditions, n (%)**	12 (13.0)	9 (19.6)	3 (6.5)	0.119
	**Respiratory failure due to septic shock, n (%)**	3 (3.3)	1 (2.2)	2 (4.3)	1.000
	**Respiratory failure due to BPD exacerbation, n (%)**	3 (3.3)	2 (4.3)	1 (2.2)	1.000
	**Respiratory failure due to asthmatic exacerbation n (%)**	2 (2.2)	1 (2.2)	1 (2.2)	1.000
**PIM 2 score**		4.3 (1.3, 9.3)^#^	5.2 (2.0, 9.5)^#^	2.5 (1.0, 9.3)^#^	0.168
**NIV before intubation, n (%)**		33 (35.9)	18 (39.1)	15 (32.6)	0.664
**Red blood cell transfusion≥ 10 ml/kg, n (%)**		38 (41.3)	21 (45.7)	17 (37.0)	0.526
**Multiple antibiotics, n (%)**		71 (77.2)	43 (93.5)	28 (60.9)	*<0*.001*
**Corticosteroids, n (%)**		36 (39.1)	15 (32.6)	21 (45.7)	0.285
**Continuous sedation on the 1st day, n (%)**		19 (20.7)	14 (30.4)	5 (10.9)	*0*.038*
**Vasoactive drug use, n (%)**		28 (30.4)	19 (41.3)	9 (19.6)	*0*.040*
**Laboratory investigations during 24 hours of admission**					
**Hemoglobin, g/dl**		10.3 (8.8, 11.7)^#^	10.3 (9.0, 11.7)^#^	10.2 (8.5, 11.7)^#^	0.993
**Hypokalemia, n (%)**		26 (28.3)	12 (26.1)	14 (30.4)	0.817
**Hypophosphatemia, n (%)**		14 (15.2)	8 (17.4)	6 (13.0)	0.773
**Hyponatremia, n (%)**		7 (7.6)	3 (6.5)	4 (8.7)	1.000
**Hypoalbuminemia, n (%)**		17 (18.5)	6 (13.0)	11 (23.9)	0.283
**Complications during 96 hours of admission**					
**VAP, n (%)**		15 (16.3)	15 (32.6)	0	*<0*.001*
**UTI, n (%)**		8 (8.7)	8 (17.4)	0	*0*.006*
**Multiple organ failure, n (%)**		14 (15.2)	7 (15.2)	7 (15.2)	1.000
**Ventilatory parameters during 24 hours of admission**					
**PIP on the first day, cmH**_**2**_ **O**		20 (16.0,22.8)^#^	20 (17, 24.3)^#^	18 (14, 22)^#^	*0*.010*
**PEEP on the first day, cmH**_**2**_ **O**		4 (3,5)^#^	5 (3.8, 5)^#^	4 (3, 5)^#^	0.112
**FiO**_**2**_ **on the first day**		0.6 (0.4, 0.6)^#^	0.60 (0.4, 0.8)^#^	0.55 (0.4, 0.6)^#^	0.151
**MAP on the first day, cmH**_**2**_ **O**		8.50 (7, 10.0)^#^	9.50 (8,10.0)^#^	8 (6.0, 9.3)^#^	*0*.017*
**LOS in hospital**		20 (11.3, 32.8)^#^	24.50 (15.8, 47.8)^#^	14.00 (7.0, 26.3)^#^	*0*.004*
**LOS in PICU**		7 (3, 12)^#^	12.0(8.8, 16)^#^	3 (1, 5)^#^	*<0*.001*
**Mortality in PICU, n (%)**		13 (14.1)	7 (15.2)	6 (13.0)	1.000

^#^ presented as median (IQR)

*: Statistically significance (*p< 0*.*05*)

**Abbreviation:** ARDS, acute respiratory distress syndrome; BMI, body mass index; BPD, bronchopulmonary dysplasia; FiO_2_, Fraction of inspired oxygen; GI, gastrointestinal disease; kg, kilogram; LOS, length of stay; MAP, mean airway pressure; NIV, non-invasive ventilation; PEEP, Positive—end expiratory pressure; PICU, pediatric intensive care unit; PIM2, pediatric index of mortality; PIP, Peak inspiratory pressure; PMV, prolonged mechanical ventilation; UTI, urinary tract infection; VAP, ventilator associated pneumonia

Multivariable logistic regression analysis identified three independent risk factors for PMV: age under 2 years (OR 2.86, 95% CI 1.04–7.84, *p = 0*.*041*), male gender (OR 3.21, 95% CI 1.15–8.94, *p = 0*.*026*), and the administration of multiple antibiotics during PICU admission (OR 7.83, 95% CI 1.87–32.78, *p = 0*.*005*) (see Table 2).

**Table 2:** Factors associated with PMV in PICU (n = 92).

Parameters	Univariable analysis		Multivariable analysis	
	OR (95% CI)	*P-value*	Adjusted OR (95%CI)	*P-value*
**Younger children under 2 years old, n (%)**	2.02 (0.88–4.63)	0.097	2.86 (1.04–7.84)	*0*.041*
**Male, n (%)**	2.92 (1.19–7.11)	*0*.019*	3.21 (1.15–8.94)	*0*.026*
**Multiple antibiotics, n (%)**	9.21 (2.48–34.21)	*0*.001*	7.83 (1.87–32.78)	*0*.005*
**Continuous sedation on the 1**^**st**^ **day of PICU admission, n (%)**	3.59 (1.17–11.01)	*0*.026*	2.77 (0.77–9.99)	0.120
**Vasoactive drug use, n (%)**	2.89 (1.14–7.37)	*0*.026*	2.54 (0.84–7.67)	0.097
**MAP on the 1**^**st**^ **day ≥ 13 cm.H**_**2**_**O, n (%)**	1.36 (0.29–6.47)	0.695	1.06 (0.17–6.57)	0.951

*: Statistically significance (*p < 0*.*05*)

**Abbreviation:** MAP Mean airway pressure; OR, odds ratio; PICU, pediatric intensive care unit; PMV, prolonged mechanical ventilation; 95% CI, 95% confidence interval

## Discussion

We report on the incidence and associated factors of PMV in a Thai pediatric institution within a publicly funded healthcare system. In our cohort, the incidence of PMV was 50%. Factors linked to PMV included younger age, male gender, and the use of multiple antibiotics.

The previous information about the incidence of PMV in pediatric patients in different PICUs varied from 2.5%-14% [10, 16, 22]. Monteverde et al. published a prospective cohort study regarding characteristics and risk factors of pediatric patients (aged 1 month-15 years) who received IMV and NIV in four medical-surgical PICUs, in Argentina from June to August, 2007 [10]. Their PMV has been defined as the need for at least 6 hours of MV per day for > 21 consecutive days. The incidence of PMV was 9%, which was calculated by using several PMV patients divided by all PICU admissions [10]. Payen et al. studied retrospectively the incidence and risk factors of PMV in PICUs in Canada from 2006–2007 [22]. Like our study, they found the incidence of PMV to be 14% (calculated by using episodes of PMV divided by all PICU admission), and PMV was defined if the duration of MV was ≥ 96 hours. Another retrospective study of pediatric patients aged 1month to 15 years who were on IMV for at least 21 days in three Brazilian PICUs has been published [16]. This study demonstrated that the incidence of PMV calculated based on the number of patients requiring PMV per 100 consecutive patients admitted into each PICU was 2.5% which was lower than our study [16]. With limited evidence to determine the definition of PMV in children [27], and different calculations of incidence of PMV as well as different pediatric patients, it causes the different incidence of PMV.

In our study, younger children under 2 years old were identified as an independent factor associated with PMV, consistent with the findings of the Liu et al. study [8]. We observed that a higher proportion of children under 2 years old in our cohort had a history of prematurity (12/46, 34.8%) compared to those aged 2 years and older (4/46, 8.7%). Prematurity can result in underdeveloped lungs, including widespread atelectasis, difficulty achieving functional residual capacity (FRC), and high chest wall compliance [28–30], which can lead to respiratory failure and necessitate MV. Additionally, prematurity may necessitate PMV, and an extended duration of initial IMV has been significantly associated with higher rates of moderate-to-severe BPD [31]. Various respiratory vulnerabilities and developmental aspects of respiratory physiology in young children, such as increased airway resistance, reduced lung volume, and decreased efficiency and endurance of respiratory muscles, contribute to a heightened risk of respiratory failure [32], which may be linked to a higher incidence of PMV.

In our study, the male gender was another factor associated with PMV. The reason for this observation remains unclear. There were some studies regarding gender differences in adults who received MV. Schwer Cl, et al. reported a retrospective study about risk factors for PMV and delayed extubation in patients undergoing surgery and found that the male gender is considered to be an independent risk factor for delayed extubation and PMV in patients undergoing surgery [33]. The reason for this observation remains unclear. However, there is growing evidence of sex-specific differences in mechanically ventilated patients. Another retrospective study in adult patients who were admitted to a general ICU showed that the length of time that women remained sedated was shorter than men. This can be inferred that women had a significantly shorter duration of MV, time to withdrawal of sedation, and ability to perform active exercises, which suggests a better response to the critical event of hospitalization [34]. However, larger, multi-center studies are required to determine whether this factor is truly linked to PMV in pediatric patients.

Furthermore, multiple antibiotics during 96 hours of admission was another independent risk factor for PMV in our cohort. Similarly, a prospective cohort study of characteristics and risk factors of pediatric patients aged 30 days to 15 years old, who received PMV in four PICUs found that factor was associated significantly with PMV was treatment with multiple antibiotics [10]. Based on our study, there was a higher incidence of VAP 32.6% (15/46) in the PMV group. Of these, 15.2% (14/46) required multiple antibiotics. Therefore, this can be inferred that multiple antibiotics were frequently required in children who were diagnosed with VAP in the PMV group [35]. Antalová et al. reported that a combination of antibiotics is required in patients with high possible antibiotic resistance and signs of sepsis and VAP. However, antibiotics are used for most patients initially required broad-spectrum therapy, followed by a narrowing and focusing of therapy as clinical and culture data [36].

In our study, continuous intravenous sedation on the first day of PICU admission, the use of vasoactive drugs, and a high MAP setting on MV were not identified as independent risk factors for PMV, which contrasts with findings from other studies [16, 22, 37, 38].

The mortality rate in our study was 14.1%, whereas other studies had mortality rates ranging from 43–48% [10, 16]. Traiber et al. reported a retrospective study to describe the characteristics of children submitted to PMV [16]. This study found that 31% of chronic comorbidity was cardiac diseases associated with PMV [16]. The main reason for MV and PICU admission was general postoperative care (10%) as well as a mortality rate of 48% [16]. Furthermore, Monteverde et al. published a prospective cohort study of pediatric patients who received PMV and demonstrated that the reason for MV were septic shock (22%) and postoperative (4%), respectively and the mortality rate in the PMV group was 43% [10]. The mortality rate in our study was less than in other studies which may be a result of different pediatric patients since CHD was excluded from our study. Moreover, the indication for IMV in our study was mainly severe pneumonia or ARDS (47.8%) and postoperative care (30.4%). According to the different indications for IMV and PICU admission, it causes different mortality rates. Some patients required tracheostomy which was higher than in other studies and the decision-making for tracheostomy after intubation was shorter than in another study [16]. The beginning of IMV until the tracheostomy is performed in adults ranging from 10 to 12 days [27, 39, 40]. However, in pediatric patients, the risks and indications to perform a tracheostomy are different compared to the adult group. The average time to insertion of a tracheostomy tube range from 4.3 to 30.4 days in pediatric patients [41–43]. However, currently, there is no consensus about the timing to perform tracheostomy in children [39–41, 44]. Given the variability in clinical practice and decision-making in PICU management as well as a family decision, results in the variation of time to tracheostomy.

Our cohort study has some significant limitations. First, the retrospective nature of this study constrained the scope of data collection. Therefore, this can cause information bias. However, the missing data was about 4.3%, which had little significance for statistical analysis. Secondly, due to the retrospective design of this study, we were unable to determine the health-related quality of life of the patients and their families after PMV. Thirdly, our study did not include children with CHD or those who had undergone cardiac surgery, as they received ICU care in a separate unit within our institution. This study reflects the experience of a single tertiary care center over one year, resulting in a small PICU sample size, which may limit the generalizability of these findings to children in other settings. Lastly, there is no standardized weaning protocol for frontline clinicians in our institution, and extubation assessment is dependent on the attending staff. Consequently, PMV might be influenced by the decision-making of the attending staff. This may limit the applicability of these findings to children in different settings. Future research should focus on key parameters that may be associated with PMV, such as the identification of pathogens in respiratory specimens, trends in sedation and muscle relaxant use, fluid overload status, neurological complications, the oxygenation index, and the use of iNO, among others. Future multi-center studies are also necessary to compare our findings with those from other institutions in order to create evidence-based clinical practice guidelines for optimal pediatric PMV management.

## Conclusions

In our study, 50% of pediatric patients underwent PMV. Contributing factors included younger age, male gender, and multiple antibiotic use within the first 96 hours of PICU admission. These findings could guide the creation of weaning protocols and therapeutic strategies to lower the incidence of PMV in the PICU. Additional large-scale, multi-center studies are needed to validate these findings and establish evidence-based clinical practice guidelines for pediatric PMV.

## Supporting information

S1 Dataset (XLSX)

## Abbreviations

ARDS: acute respiratory distress syndrome
BPD: bronchopulmonary dysplasia
BW: body weight
CDC: The Centers for Disease Control and Prevention
CFU: Colony-forming unit
CHD: congenital heart disease
CLABSI: central line- associated bloodstream infection
CRRT: continuous replacement therapy
dl: deciliter
DSI: daily sedation interruption
FiO_2_: fraction of inspired oxygen
FRC: functional residual capacity
g: grams
GI: gastrointestinal disease
HPF: High-power field
ICD: International Classification of Disease
ICU: intensive care unit
IMV: invasive mechanical ventilation
IQR: interquartile range
kg: kilogram
L: liter
LCBI: laboratory confirmed bloodstream infection
LOS: length of stay
MAP: mean arterial pressure
mg: milligram
mm^3^: cubic millimeter
mmol: millimole
MV: minute ventilation
NIV: non-invasive ventilation
OR: odds ratio
PEEP: positive end expiratory pressure
PICU: pediatric intensive care unit
PIP: peak inspiratory pressure
PMV: prolonged mechanical ventilation
SD: standard deviation
UTI: urinary tract infection
VAP: ventilator associated pneumonia
VIF: variance inflation factor
WBC: white blood cell count
